# The Rheology of the Carotid Sinus: A Path Toward Bioinspired Intervention

**DOI:** 10.3389/fbioe.2021.678048

**Published:** 2021-06-10

**Authors:** Andrew Iskander, Coskun Bilgi, Rotem Naftalovich, Ilker Hacihaliloglu, Tolga Berkman, Daniel Naftalovich, Niema Pahlevan

**Affiliations:** ^1^Department of Anesthesiology, Westchester Medical Center, New York Medical College, Valhalla, NY, United States; ^2^Department of Aerospace and Mechanical Engineering, University of Southern California, Los Angeles, CA, United States; ^3^Department of Anesthesiology, New Jersey Medical School, University Hospital, Rutgers University, Newark, NJ, United States; ^4^Medical Corps of the U.S. Army, U.S. Army Medical Department, Fort Sam Houston, San Antonio, TX, United States; ^5^Department of Biomedical Engineering, Rutgers School of Engineering, Rutgers University, Piscataway, NJ, United States; ^6^Department of Computational and Mathematical Sciences, California Institute of Technology, Pasadena, CA, United States; ^7^Keck School of Medicine, University of Southern California, Los Angeles, CA, United States

**Keywords:** baroreceptor, blood flow, viscosity, PIEZO receptor, carotid sinus

## Abstract

The association between blood viscosity and pathological conditions involving a number of organ systems is well known. However, how the body measures and maintains appropriate blood viscosity is not well-described. The literature endorsing the function of the carotid sinus as a site of baroreception can be traced back to some of the earliest descriptions of digital pressure on the neck producing a drop in blood delivery to the brain. For the last 30 years, improved computational fluid dynamic (CFD) simulations of blood flow within the carotid sinus have demonstrated a more nuanced understanding of the changes in the region as it relates to changes in conventional metrics of cardiovascular function, including blood pressure. We suggest that the unique flow patterns within the carotid sinus may make it an ideal site to transduce flow data that can, in turn, enable real-time measurement of blood *viscosity*. The recent characterization of the PIEZO receptor family in the sinus vessel wall may provide a biological basis for this characterization. When coupled with other biomarkers of cardiovascular performance and descriptions of the blood rheology unique to the sinus region, this represents a novel venue for bioinspired design that may enable end-users to manipulate and optimize blood flow.

## Descriptions of the Carotid Sinus Physiology Throughout History

*“I have gained the conviction through repeated and careful observations, that the slowing of the pulse when pressure is applied on the carotid is a frequent finding, in healthy, as well as in sick persons.”* (Huth and Murray, 2006).

-Heinrich Irenaeus Quincke

### The Carotid Bifurcation Before the “Carotid Sinus”

In 1875 Quincke, a German physician responsible for introducing the lumbar puncture, spoke of the commonly held association between compression of the carotid bifurcation and decreased cardiac output. The association between the very immediate effects of compression of the vessels in the neck and a drop in global blood flow has been noted for a very long time. (The Greek word for “stupefy”, “karos”, provides the basis for our modern “carotid.”). Indeed, Rufus of Ephesus, circa 50 AD, described the almost immediate change in blood flow and mentation when the vessels in the neck were compressed (Munster et al., 2016). For the 1,800 years that ensued, the notion that there was a profound slowing of the heart rate with compression of the neck vessels was attributed, somewhat correctly, to the nerves adjacent to the carotid (Glick and Covell, 1968; Persson and Kirchheim, 1991). As the heart rate was noted to decrease with manipulation of the dilated portion of the internal carotid, the vagus nerve was taken for granted up until the 1920s as being responsible for transmitting this signal to the autonomic nervous system.

### The Current Paradigm of the Carotid Sinus as a Baroreceptor

It was Heinrich Ewald Hering in 1924 (Zimmer, 2004) who, through a series of experiments with over a hundred dogs, rationalized the anatomy and function of the carotid sinus and its nerve as it is currently understood. He observed that internal direct mechanical application of a pressure stimulus to the carotid sinus region using a brass probe had similar effects as direct electrical stimulation to the sinus. He realized that the nerve found to insert in the adventitia was distinct from the vagus nerve. Furthermore, he demonstrated that the electrical stimulation of this nerve also elicited the same effect as a clamp applied to the sinus–a reduction of heart rate and vasodilation with a resultant drop in blood pressure. The presumption is that this “vagal” parasympathetic effect does not originate in the vagus nerve. The role of the carotid sinus in global perfusion was further supported by Hering’s observation that systemic blood pressure reduced with increased pressure within the sinus itself (Persson and Kirchheim, 1991). His pursuit of a nerve responsible for mediating these observed cardiovascular responses led him to the eponymous Hering’s nerve, also known as the carotid sinus nerve, and helped complete our contemporary understanding of baroreception.

### The Clinical Need for a Novel Biomarker of Blood Viscosity

The predictive relevance of blood viscosity in the literature has been suspected to be intrinsically germane to many disease processes. Types of problems where blood viscosity appears to play some pathophysiologic role include immunologic diseases (Gudmundsson et al., 1993), inflammatory diseases (Nwose, 2010), hemolytic anemias (Bowers et al., 2013, 2018; Kucukal et al., 2020), hearing loss (Hildesheimer et al., 1990; Garcia Callejo et al., 2006), diabetes (Nakanishi et al., 2004; Richards and Nwose, 2010; Schiapaccassa et al., 2019), renal disorders (Jung et al., 2017), sickle cell disease (Klug et al., 1974), and cerebrovascular disease (Song et al., 2017). However, the association between cardiovascular disease and blood viscosity has been looked at most extensively throughout the literature (Lowe, 1992; Kenyeres et al., 2008; Chevalier et al., 2013; Buyan et al., 2017; Celik et al., 2017; Peters et al., 2017; Sloop et al., 2018; Cekirdekci and Bugan, 2020; Engin and Guvenc, 2020).

In an attempt to determine whether increased blood viscosity has any predictive value as a biomarker in the setting of cardiovascular disease, Peters (Peters et al., 2017) compiled blood viscosity data from the Scottish Heart Health Extended Cohort (SHHEC). The SHEEC (Woodward et al., 2007) included participants without known cardiovascular disease recruited across Scotland from two different cohorts of men and women, one group from 1984–1987 and the other from north Glasgow in 1989, 1992, and 1995. For this prospective study, they compiled the data in the hopes of creating an ASSIGN (Assessing Cardiovascular Risk Using SIGN Guidelines) cardiovascular morbidity and mortality risk score. By taking venous blood samples, they calculated the relative blood and plasma viscosities. They uncovered a statistically significant association between cardiovascular and all-cause risk with blood viscosity, particularly plasma viscosity. More importantly, the study demonstrated that even when controlling for the normal increases in viscosity associated with age, sex, and other known cardiovascular risks, viscosity had predictive value in ultimate scoring for mortality risk.

In another study (Skretteberg et al., 2010) from Sweden, patients with no known cardiovascular disease were recruited into a study whereby hematocrit was related to long-term outcomes. After controlling for other known causes of cardiovascular mortality, they found an association between elevated sedimentation rate – a broad measure of inflammation – and elevated hematocrit. Remarkably, the association remained nearly as robust 26 years after enrollment as it did 10 years after enrollment. This study further corroborates the notion that blood viscosity may have predictive value independent of other known causes of cardiovascular mortality. The authors conclude that their findings support the theory that “hematocrit, plasma viscosity, and inflammation may increase… morbidity and mortality by promoting thrombotic complications and… atherosclerosis.” Based on its robust and long-lasting association, the authors argue that blood viscosity should be an independent prognostic indicator for cardiovascular events and mortality.

While increasing plasma viscosity leading to increased end-organ dysfunction may make intuitive sense, the question arises: “How does the body detect ‘optimal’ blood viscosity?” Is there an apparatus that can be described as the body’s own viscometer? Is that structure able, by virtue of its structure and function, to shed light on the rheology of global blood flow? Is this structure involved in the function of other organs whose functions titrate the fluid components involved in comprising blood viscosity?

## The Carotid Sinus as a Viscometer

The role of the carotid sinus as a pressure sensor is well-known (Andani and Khan, 2020). The present review discusses the intrinsic function of the carotid sinus as it relates to blood rheology and to the microanatomical apparatus responsible for initiation of its action. This review does not focus on the extensive and important work by many in the field of endothelial mechanobiology (a field which owes a great deal to Peter F. Davies for much of our understanding of the mechanics of endothelial transduction). This review is not a comprehensive review of the entire downstream pathway that follows after sinus activation – a pathway that we believe involves the vagus nerve and further neuromodulation by the central nervous system. Here, we aim to present aspects of the structure and function of the carotid sinus that may support its role as a blood viscometer.

As applied physiologists, clinicians manipulate cardiovascular homeostasis on a gross scale. For example, during carotid endarterectomy surgeries, glycopyrrolate is often given at the time of anesthetic induction to attenuate the reflex bradycardia that often ensues after carotid stent deployment. Despite our familiarity with this mechanism, clinicians often overlook the blood flow characteristics within the carotid sinus that may have a role in maintaining hemodynamics. This review presents recent improvements in our understanding of the blood rheology and microphysiology of the carotid sinus to enhance the clinician’s working knowledge. Considering the function of the carotid sinus and its possible role as a sensory organ may provide the basis for bioinspired design of devices that better enable clinicians to read, interpret, and manage blood viscosity.

## The Unique Flow and Shear Stress Characteristics of the Carotid Sinus

The salience of carotid ultrasound to anesthetic management is increasing. In many centers, carotid Doppler studies are part of the preoperative workup for many major surgeries. For patients undergoing cardiac surgery, carotid ultrasound is a cost-effective, non-invasive screening tool that most anesthesiologists probably take for granted and view as having somewhat of a distant relevance to patient evaluation. However, data obtained by carotid Doppler is now used to guide blood flow (Weber et al., 2016) and stratify delirium risk (Bernardi et al., 2019) in the immediate postoperative period, as well as for a longer-term perspective relating to post-operative cognitive dysfunction (Elias et al., 2019).

### The Flow Dynamics Within the Carotid Sinus

**FIGURE 1:**
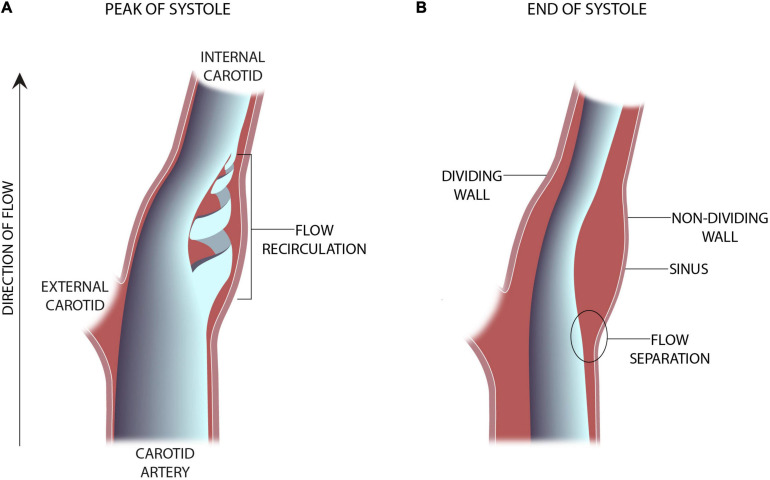
Schematic representation of an in-vitro experimental model of the carotid sinus (Ku and Giddens, 1983). **(A)** The presence of vortical or flow recirculation during the peak of systole results in sheer stress “impulses” against the sinus wall. **(B)** The anatomy of the sinus causes flow separation from the non-dividing wall resulting in a region of low wall shear stress.

**FIGURE 2:**
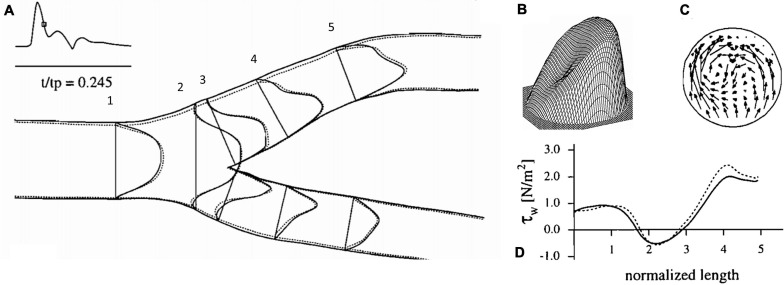
Flow dynamics inside the carotid artery at deceleration, **(A)** axial velocity profiles, **(B)** three-dimensional representation of axial velocity profile at 4, **(C)** representation of circulatory flow vectors at 4, **(D)** wall shear stress values along the outer carotid wall (Karner et al., 1999).

The advent of clinically useful ultrasound examinations of the carotids created a need for a greater in-depth appreciation for the hemodynamic uniqueness of the sinus region. The very specific location of the sinus, immediately distal to the carotid bifurcation at the internal carotid artery inlet and above the level of the heart, lends its flow characteristics to vary over the cardiac cycle. The first comprehensive descriptions of the unique flow within the carotid sinus were first consolidated in 1983 (Ku and Giddens, 1983). The sinus is unique for the region of “flow separation” from the non-dividing wall at the origin of the internal carotid (Ku et al., 1985a,b; Ku and Giddens, 1987). Firstly, there is an effective coalescing of the flow streamlines in the origin of the internal carotid that results from the flow separation away from the non-dividing wall (Figure 1). Essentially, the flow vectors orient toward the carotid dividing wall and “make room” for the region of swirling, or recirculation, within the dilated region (Figure 2; Karner et al., 1999). This results in constant flow shear against the dividing wall throughout the cardiac cycle. Secondly, this high-velocity flow (Figure 2A) at the dividing wall leads to persistent reduced shear stress at the non-dividing wall of the sinus where the carotid sinus inserts into the adventitia. Lastly, the vortex of the fluid within the sinus, Figures 2B,C, causes alternating levels of shear stress “impulses” that change in the magnitude and polarity over the cardiac cycle. Direction change of wall shear stress (WSS) can be observed in Figure 2D, which results in oscillations of stress throughout the cardiac cycle. Interestingly, it is the oscillations from WSS that have long been accepted as the inciting factor for atheroma formation in this portion of the internal carotid (de Vecchis et al., 2010; Hirata et al., 2011; Leisser et al., 2015; Saba et al., 2015). It is therefore not surprising that the carotid atherosclerotic plaque is often found in the sinus (Gulevskaia et al., 2007).

The advent of open-source platforms for computational fluid dynamic (CFD) simulations led to a more quantitative, data-driven understanding of the flow characteristics of the carotid vessels, especially of the shear stress-related flow patterns within the sinus (Marshall et al., 2004; Milos et al., 2011; Dong et al., 2013; Zhang et al., 2013; Sui et al., 2015; Cibis et al., 2016; Guo et al., 2018; Xu et al., 2018; Zhang D. et al., 2018; Dai et al., 2019; Iskander et al., 2020). Enabling visualization of flow makes this information more translatable to clinical practice by emphasizing the possible role of the shear-thinning aspects of blood flow. Patient-specific CFD studies suggest that the non-Newtonian behavior–including shear-thinning–of blood is negligible in large arteries (Lee and Steinman, 2007; Arzani, 2018). However, recent studies on simplified dilation geometries, such as is seen in the sinus, reveals the significant differences in WSS-related parameters seen with even small changes in the viscoelastic and shear-thinning behavior of blood (Bilgi and Atalik, 2019, 2020). Furthermore, experiments on Fontan hemodynamics highlight that neglecting non-Newtonian behavior like shear-thinning can produce significant errors and misinterpretation of the hemodynamics (Cheng et al., 2018a,b, 2019; Wei H. et al., 2020; Wei Z. et al., 2020). These studies demonstrate the important role of shear-thinning relevant to specific clinical problems such as Fontan flows and aneurysms.

The key parameter in triggering non-Newtonian effects is the shear rate, which depends on local fluid dynamics of the blood at any specific location. Shear-thinning is a property observed during low shear rate (low velocity gradient) in which the apparent viscosity increases as the velocity gradient (shear rate) decreases. Alternatively, at high shear rate, the apparent viscosity decreases until it reaches a constant viscosity value and it behaves Newtonian. Due to the Fahraeus-Lindqvist effect, the apparent viscosity further decreases in smaller vessels with diameters between 30 to 300 μm (Truskey et al., 2004). Ku and Giddens (1983) also demonstrated that in the sinus region, the wall is under *less* WSS throughout the cardiac cycle. This occurs since the sinus wall is drawn away from the opposing streamlined flow whose vector orients more toward the dividing wall than toward the sinus. Lee and Steinman (2007) developed a CFD model of the bifurcation and related changes in hematocrit to changes in the sinus region. In agreement with other models of the sinus, this area of low WSS corresponds to a zone of increased shear oscillation resulting from the vortical swirling characteristic of that region. They demonstrated that changes in the modeled root mean square WSS of 5–15% corresponded to hematocrit changes of 20% in the same direction. In short, they showed that the shear-thinning aspect of blood can be considered when modeling blood flow, and that this aspect is relatable to the velocity and hematocrit of blood, two immediately measurable clinical parameters.

**FIGURE 3:**
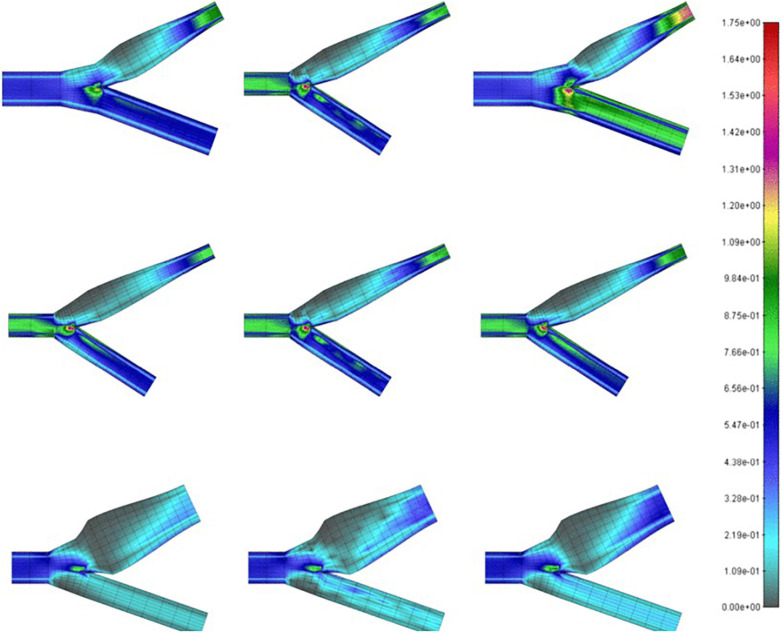
Wall shear stress (in Pa) on carotid bifurcations of differing geometries obtained by CFD (left), MPL (middle), k-NN (right) algorithms (Milos et al., 2011).

Milos (Milos et al., 2011), looked at the CFD models of over 1,400 carotid arteries using data mining methods and began to draw generalizations about carotid geometry, blood viscosity, velocity, density, and several other clinically relevant parameters which correlates to the region of recirculation in the sinus. They aimed to determine the feasibility and logistics of using different machine learning models to associate local WSS and the region of recirculation with precision. Figure 3 shows a comparison of the WSS values of conventional CFD with their multilayer perceptron neural network (MPL) and k-nearest neighbors (k-NN) algorithms. They ultimately demonstrated the availability of clinically useful machine learning algorithms that can accurately predict the flow and stress fields by using anatomical data from ultrasound imaging. The required parameters like sinus diameter, sinus length, angle of the internal carotid relative to the common carotid, blood density, and velocity and clinically relevant proxies for hematocrit and cardiac output, can be easily and non-invasively collected from a patient.

Extracting data from simulations has also led to parameters previously only available from Doppler data (Gates et al., 2018; Zhang B. et al., 2018). In the work by Zhang B. et al. (2018), they obtained ultrasounds of two groups of fifty patients. One group had “normal” intimal-media thickness ratios and the other was “thickened.” By taking dimensional measurements in and around the carotid sinus, they used conventional Doppler data to calculate values previously only available in a simulated setting: blood viscosity, WSS, and velocity gradients within the carotid sinus. When these parameters were correlated with blood pressure and cardiac output, in patients with higher blood pressures, the region of greatest WSS was smaller in volume and made contact with less area of the carotid sinus wall along the longitudinal orientation.

A more recent advancement is Vector Flow Imaging (VFI) which utilizes the Doppler data to visually construct the flow lines within the sinus. VFI is a robust method for obtaining 2D images of the velocity vector profiles well-suited for areas with vortical flow such as the sinus. The real advantage to this method is that the forward (i.e., toward the brain) vs. reverse flow can be depicted simultaneously, allowing for measurement of the degree of turbulence within the region of recirculation (Goddi et al., 2017). As VFI finds greater availability, more precise quantification of the non-laminar flow in the sinus is expected.

## Mechanotransduction by the Carotid Sinus

### The Role of Shear Stress Mechanotransduction

The unique blood flow at the sinus creates flow patterns and shear forces that are transduced to the central nervous system. The term *mechanotransduction* refers to the transmission of a physical extracellular input or trigger to a cellular output. The physical forces that lead to these biological responses include direct cellular contact with shear forces, changes in transmembrane voltage, and mechanical stretch. The pervasive role of mechanotransduction in so many *in vivo* processes cannot be overstated. In the literature, there is a role for mechanotransduction in voluntary urination (Mukhopadhyay and Stowers, 2020), guiding cell proliferation during embryological development (Wozniak and Chen, 2009), cardiomyocyte shape and function (McCain and Parker, 2011), renal tubule function (Weinbaum et al., 2011), touch (Sanzeni et al., 2019), regulation of vascular smooth muscle tone (Sazonova et al., 2015), pulmonary smooth muscle tone (Noble et al., 2014), pain (Feng and Guo, 2019), and many others. As applied physiologists, the modulation of these mechanisms is likely to have an increased role in the management of patients undergoing anesthesia. A classic example is utilizing mechanical ventilation settings that mitigate lung injury from shear forces due to positive pressure ventilation (Jamaati et al., 2016).

Amongst the physical phenomena that serve as the triggers for mechanotransduction in endothelial cells, the most important is shear stress. Shear stress is defined as the force created when two adjacent parcels of fluid are traveling adjacent to each other at different velocities. The force created between the parcels by this difference in speed at the point of contact between them is shear. In the case of simple laminar flow (i.e., blood flow direction parallels the vessel wall), the blood velocity profile is fastest at the center of the lumen, and the velocity decreases in a series of concentric circles approaching zero flow when in contact with the endothelial surface.

The local environment of the sinus leads to a region of recirculation which makes one ponder the relationship between its structure and function. The structure of the sinus is a dilation just distal to a bifurcation. This dilation creates a sudden expansion, and it leads to disturbance of the flow and vortex formation (Nguyen et al., 2008). This geometry, together with the cardiac cycle, leads to a pulsatile wash-out of the carotid stretch that accompanies each systolic pressure peak with every beat (Ku and Giddens, 1983). In other words, the sinus, due to the dilation geometry, is also experiencing a non-uniform stress field with pulsatile flow (Bilgi and Atalik, 2020). These stress fields lead to the development of recirculation regions inside the sinus. The types of forces that act on vascular endothelium are broadly thought of as WSS or circumferential stretch (Lu and Kassab, 2011). Whereas circumferential stretch reflects blood pressure, WSS depends on fluid properties and flow conditions, *and it is highly affected by viscosity* (Lee et al., 2020). Therefore, the unique presence of such stress fields suggests that transduction at the sinus may be biased toward shear stress as it carries more information on the overall hemodynamics than just pulsatile pressure.

The characteristics of the shear stress patterns specific to the sinus have been studied extensively since these patterns are believed to underlie the pathophysiology for atherogenesis in the carotid. The regions noted to have the highest incidence are often associated with the *lowest* WSS (Zhang et al., 2012). The region of low WSS may *independently* cause intimal-medial thickening (Irace et al., 2004; Liu et al., 2016; Zhang H. et al., 2018). Also it is thought to be a contributing nidus in the inflammatory cascade that ultimately leads to atherogenesis, as endothelial cells at the arterial vessels require WSS values of ≈2 Pa to avoid morphological changes (Malek et al., 1999). As a result, there are many efforts to utilize non-invasive quantification of WSS aimed at identifying patients who will develop carotid plaques (Katakami, 2016).

**FIGURE 4:**
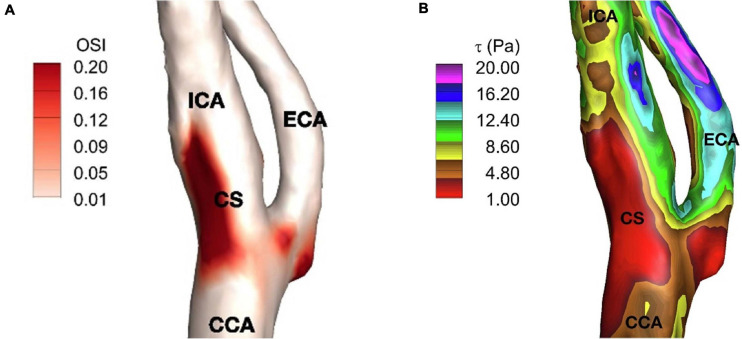
**(A)** Time-averaged wall shear stress**, (B)** oscillatory shear index on carotid artery. ICA, internal carotid artery; ECA, external carotid artery; CCA, common carotid artery; and CS, carotid sinus (Iskander et al., 2020).

The region of the lowest WSS is a consequence of the disruption of laminar flow in the sinus described by Ku. This area of lowest WSS correlates with the region of highest shear oscillation (Zhang et al., 2012). To describe this, Ku posited the Oscillatory Shear Index (OSI) (Ku and Giddens, 1983) metric to describe the degree of the WSS direction persistency during a cardiac cycle. Essentially, the OSI quantifies the amount of WSS deflection from the average over a cardiac cycle, due to flow disturbance, and OSI is reported between 0 and 0.5, where 0 denotes no change in the vector direction. In an idealized Y-shaped carotid bifurcation model, OSI peak corresponded to the region of greatest intimal thickening in the inner and outer wall of the sinus. However, there was a weaker correlation along the sides of the sinus, where plaque development still occurs. To Ku’s model, Ding et al. (2001) utilized a “tuning fork” shaped model more representative of actual carotid angiograms. He found that high OSI (>0.20) also correlated well with regions of the recirculation zones inside the carotid sinus and at the side-walls. As expected, a more anatomically realistic carotid model better reflected the accompanying sinus flow patterns. Furthermore, it demonstrated that flow changes throughout the cardiac cycle correspond to specific oscillation patterns in the low WSS region. The anatomy of the carotid sinus creates local secondary flows that “enhances the pulsation of WSS under pulsatile conditions” such as when the heart is beating and hence is a site well-suited for flow transduction. A patient-specific study supporting the relation between low WSS and high OSI regions can be seen in Figure 4. Here, the reader can note the discreet overlap between the region of lowest WSS and the region of most apparent shear oscillation. In that study, this region of low WSS may enhance the ability to detect and transduce the smaller oscillatory shear forces that result from recirculation. The low WSS environment makes it EASIER to transmit the oscillatory forces through the thinner medial layers where the carotid sinus nerve inserts (Porzionato et al., 2019).

### The Role of PIEZO Receptors in Mechanotransduction of Flow

The roles of the carotid sinus apparatus in various homeostatic functions such as “cross-talk” (Wilson et al., 1990; Chen et al., 2007) between the cardiopulmonary and arterial reflexes, renal control of free water (Barger et al., 1984; Ouisuwan and Buranakarl, 2005), and regulation of vessel capacitance via smooth muscle tone (Risoe et al., 1994) suggest that mechanotransduction of blood flow in the sinus region plays a role in these processes. The breadth of functions underpinned by blood flow mechanotransduction across organ systems has led to increased interest in the relationship between shear force-gated receptors and blood rheology. An important family of shear-force gated channels are the PIEZO receptors. In vertebrates, this group is comprised only of the PIEZO1 and PEIZO2 receptors (Coste, 2012). The PIEZO1 receptor is involved in endothelial function and development (Li et al., 2014; Rode et al., 2017) as well as cardiovascular homeostasis (Rode et al., 2017). PIEZO2 receptors have a role in the function of the somatosensory apparatus involved in proprioception (Yang et al., 2016), pain sensation (Bai et al., 2017), and possibly as a coreceptor with PIEZO1 in the carotid sinus (Zeng et al., 2018).

To further support the basis of the role of the PIEZO receptors in a model of cellular transduction of mechanical stimulus, Coste et al. (2010) determined the numbers of PIEZO-containing cells in adult mice organs by mRNA quantitative polymerase chain reaction. Importantly, the average number of detected PIEZO2 cells shown in Table 1 are markedly increased for the cells that project from the dorsal root ganglia where mechanosenstive neurons originate and project to blood vessels in order to maintain vasomotor tone as shown by the presence of 478 PIEZO2 cells out of a total 2391 cells. The relative number of PIEZO cells are benchmarked on the assumption that bladder tissue has the same number of PIEZO1 cells as PIEZO2 cells. When the PIEZO2 cells were essentially deactivated, the dorsal root ganglion (DRG) cells were rendered insensitive to mechanical stimuli. Furthermore, when the PIEZO 1 and 2 receptors were *over*-expressed, the response to mechanical stimuli was increased exponentially. The origin of these neurons in a known mechanosensitive region of the DRG is consistent with previous studies (Coleridge and Coleridge, 1980; Westcott and Segal, 2013). Therefore, they concluded that the PIEZO receptors “are both necessary and sufficient” for mechanotransduction for cells in which they are expressed.

**TABLE 1:** PIEZO-containing cell numbers found in adult mice organs (Coste et al., 2010).

**Organ**	**PIEZO1 Cells**	**PIEZO2 Cells**
Bladder	206	206
Brain	23	9
Cerebellum	8	9
Colon	69	66
Dorsal Root Ganglia	13	478
Heart	15	6
Kidney	74	13
Lung	407	506
Skeletal Muscle	13	6
Skin	165	16
Small Intestine	25	19
Stomach	43	35

### PIEZO Receptor and Shear Stress From Blood Flow

There is increasing emphasis on the fundamental role of the PEIZO1 receptor in health and disease. In the commentary by Li et al. (2015) titled *Endothelial Piezo1: life depends on it*, he makes the argument that cation influx through the receptor results from shear force outside of the cell and leads to membrane tension proximal to the PIEZO channel. This triggers cation influx through the receptor into the cell. The resulting action depends on the cell in question. For example, if the relevant cell is a red blood cell, then the action may be to trigger downstream pathways meant to maintain the appropriate hydration (Cahalan et al., 2015) and turgidity of the cell, or the amount of iron turn-over from red blood cell turnover (Andolfo et al., 2020).

Similarly, in the case of the endothelial cell, the PIEZO1 receptor may enable the endothelium to serve its role as both a responder to and shaper of blood flow necessary for development and function throughout life. In a mouse model with mutated PIEZO1 activity (Li et al., 2015), the alignment of endothelial cells needed for vascular maturation was aberrant. In these embryos, the heart is developed and beating and the endothelial cells are present, yet are unable to align themselves to create mature vasculature in the direction of blood flow, leading to embryonic lethality. Without the maturation of major vessels, the development of downstream organs cannot complete. In adulthood, the alignment of endothelial cells may offer protection against atherosclerosis by reducing the local atherogenic effects of disturbed flow (Coleman et al., 2020). The alignment of endothelial cells, as mediated by mechanisms including the PIEZO protein within the cell membrane, supports the notion that the goal of transducing shear stress is highly dependent on the time and place of the cell in question.

**FIGURE 5:**
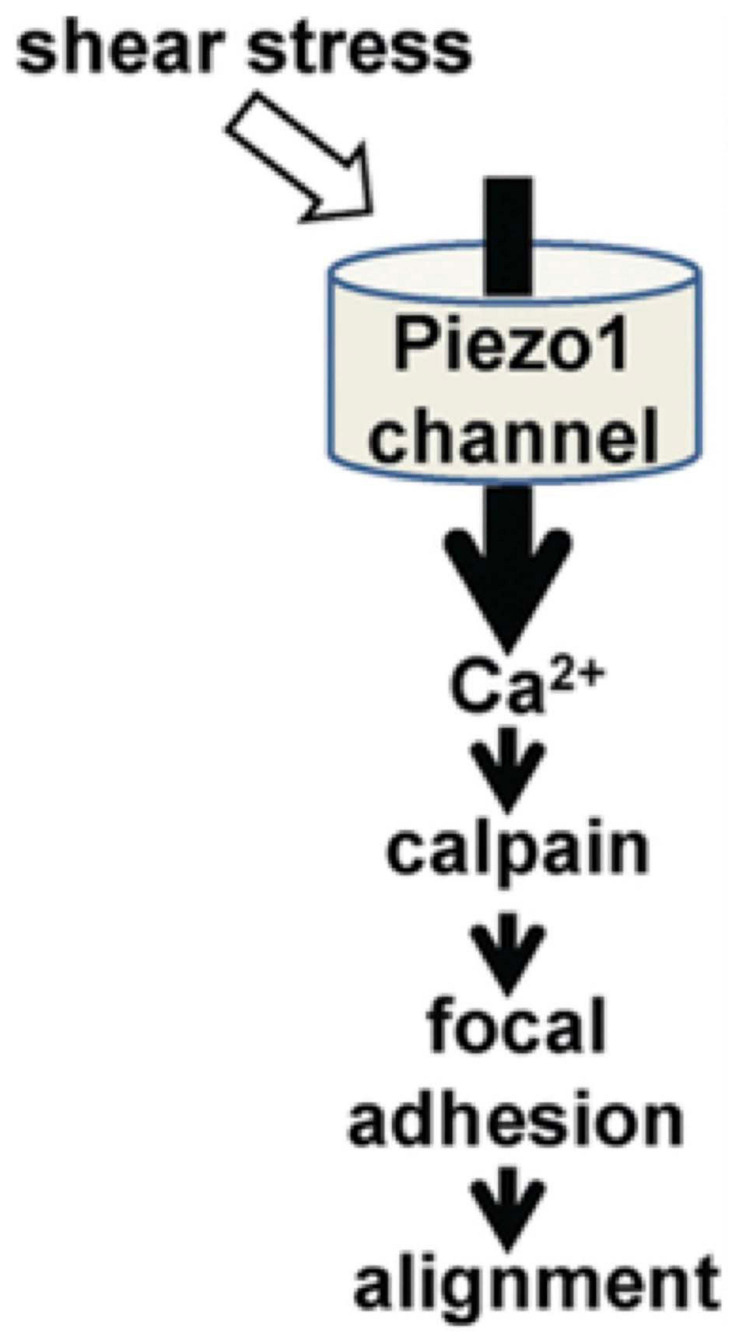
The role of PIEZO1 receptors in alignment of endothelial cells during cardiovascular embryological development (Li et al., 2014).

When detecting regions of locally created secondary flows, the PIEZO receptors are well suited to detecting endothelial flow data (Murthy et al., 2017; Douguet et al., 2019). In the work by Li et al. (2014), they demonstrate that the PIEZO1 receptors mediate shear stress-related events in human and mouse embryonic endothelial cells (Figure 5). In the *in vitro* setting, the human endothelial cells deficient in PIEZO activity were unable to align themselves in the direction of an applied shear force as seen *in vivo*. They first attached Green Fluorescent Protein to PIEZO1 proteins and found that they aggregated near the apical lamellipodia of the endothelial cells. The cells that were +/+ for the PEIZO1 genotype linearly aligned in the direction of the applied shear force. Those that were +/− were aligned in a cobble-stone fashion, and those that were −/− demonstrated no alignment. Furthermore, they demonstrated that the lack of piezo activity eliminated shear stress-induced entry of Ca^2+^ into the human endothelial cells entirely. They isolated human embryonic kidney cells that lacked PIEZO1. It was only after adding exogenous PIEZO1 activity that Ca^2+^ entry was seen in these cells. This supports the hypothesis that the PIEZO1 receptor has an important role in the detection and cellular response to shear stress.

The pivotal role of the PIEZO receptor in the detection of endothelial flow data rather than blood pressure, *per se*, is demonstrated when considering how vasculature will remodel in response to chronic changes. In the study by Retailleau et al. (2015), a murine model of PIEZO knockout was developed. Importantly, they found that the PIEZO1 receptors were located primarily in the media of the smooth muscle portion of the smaller diameter arteries, particularly the cutaneous caudal artery and the cerebral arteries, but not the larger diameter arteries including the renal artery and the aorta. In the PIEZO knockout mice where both alleles for the receptor were absent, all stretch-activated channel activity (where a patch-clamp is applied to the cell membrane as a shear stimulus) was absent. This suggests an important role for the PIEZO1 in vessel myocyte mechanotransduction. But when vasoactive substances were used, they found that the PIEZO knockout tissue from the caudal and cerebral arteries did not require the receptor to respond to vaso-constricting or vaso-dilating drugs. They did find that in the small diameter-arterial smooth muscle tissue, the PIEZO receptor was a necessary requirement to respond to the patch clamp stimulus, however. In order to examine smooth muscle remodeling in a model of hypertension, their model used an infusion of angiotensin II (AT II) infusion versus a saline as a normotensive control. They found that the arterial diameter, wall thickness, and cross-sectional area (CSA) was unchanged in the PIEZO-absent mice under normotensive conditions. In the knockout mice who underwent AT II infusions, there was a significant decrease in diameter, thickness and in CSA. Finally, they used cells where “mechanoprotection” was removed in which the actin cross-linking element Filament A (FlnA) was deleted in vascular smooth muscle. Those “unprotected” cells with the FlnA deletion resulted in PIEZO receptors that were open even without hypertension–essentially reducing the shear stimulus threshold needed for them to open–demonstrating that remodeling of smooth muscle occurred in the caudal artery without hypertension and only with activated PIEZO receptors. Just replacing one of the PIEZO alleles in these unprotected cells was enough to reverse the increase in wall thickness seen from hypertension or the removal of the FlnA. This further supported the notion that the PIEZO receptor is central to the transduction and endothelial response to shear stress and not necessarily to blood pressure.

In addition to the possible role of the PIEZO receptor in vascular remodeling, elucidating the possible role of the PIEZO receptor in the proper functioning of the endothelium may suggest a line of inquiry aimed at examining the pathophysiology of atherosclerotic disease in the carotid sinus that results from the blood flow patterns unique to it. In the comprehensive review by Gimbrone, they discuss studies that compared atheroprone geometries such as the carotid sinus with its “oscillatory” flow patterns to atheroprotective geometries such as the distal internal carotid that have more consistent laminar flow (Gimbrone and Garcia-Cardena, 2016). In the region of atheroprone endothelium such as in the sinus, flow appears to demonstrate an “absence of undisturbed laminar shear stresses”. Just upstream, however, endothelium in the distal internal carotid demonstrated upregulation of those factors associated with an atheroprotective phenotype, particularly of Kruppel-like Factor 2 (KLF2). Importantly, KLF2 has also been demonstrated to regulate production of vasoactive substances used to mediate locally mediated vasomotor tone such as nitric oxide. This locally mediated sensing and responding to shear force is essential to the role of PIEZO channels in sensing of cardiovascular force as described by Li et al. (2014) More specifically, it appears that the PIEZO receptors could be the primary players in coupling endothelial response to regulation of blood flow. As such, when PIEZO genes were disrupted, the endothelial response to increased blood flow appears to be diminished (Beech and Kalli, 2019). Given the central role of the PIEZO in sensing shear forces and the apparent importance of shear forces in atheroma prohibition and formation, PIEZO dysfunction may have a role in the development of the atheroprone phenotype.

The activation physics of the PIEZO receptor is also particularly well-suited to the type of flow unique to the sinus region described above. The recirculatory region results in secondary flows that repeatedly cause shear oscillation patterns that create a cohort of vibrations detected at the endothelium (Ku et al., 1985b). This creates a composite picture of the blood flow that reflects the particular rheological state of blood for a given cardiac output. Factors that change the oscillatory footprint of blood in the sinus region, including viscosity, blood pressure, and temperature, may only exhibit subtle changes in corresponding shear stress peaks and troughs from beat to beat. The PIEZO1 receptors have the distinctive feature of a particularly short inactivation time (Zheng et al., 2019). This is due to what Zheng et al. (2019) describes as the physical constriction within the lumen of the receptor tubule and a hydrophobic layer across the pore. This yields inactivation kinetics that are incredibly fast with time to cessation of activation of the receptor in as little as 50 ms (Wu et al., 2017b). Indeed, disease may result from slower time to inactivation (Demolombe et al., 2013). Such inactivation kinetics having few receptor channels open at a given time leads to a “temporal frequency filtering” phenomenon in which repeated vibrational stimuli can be transduced with high precision by filtering out background frequencies (Lewis and Grandl, 2015; Lewis et al., 2017). This may explain the unique suitability of the PIEZO receptor to reacting to infinitesimally small, discrete changes in the shear-force waveform in a region of vortical flow as in the carotid sinus.

### PIEZO and the Effect on Red Blood Cell Morphology

The role of the PIEZO1 receptor in blood rheology may be seen in the effects on the red blood cells themselves when the receptor function is altered. Blood viscosity and other flow parameters change with blood temperature, local microenvironments both inside and outside the cell, iron and hemoglobin state, as well as the size of the vessel and flow rate (Dupire et al., 2012). A major determinant of the intrinsic ability of a red blood cell, whose diameter ranges from 6–8 μm, to sufficiently contort itself through capillaries around 5 μm in diameter, Godwin et al. (2020) is intracellular hydration status.

The possible role of the PIEZO receptor in blood flow homeostasis is supported by PIEZO1 mutations that lead to erythrocyte changes. In the work by Cahalan (Cahalan et al., 2015), they demonstrate a relationship between PIEZO1 function and appropriate hydration of red blood cells. They showed that mechanical force applied to red blood cells by a pipette initiated entry of Ca^2+^ into cells through the PIEZO1 channel, leading to osmotic changes. Furthermore, the red blood cells of PIEZO1-deficient mice were overhydrated, more fragile, and underwent greater splenic sequestration. This suggests a role for shear forces acting on the red blood cells themselves in maintaining rheological homeostasis. This further supports the notion that PIEZO channels represent a major means by which shear forces are transduced into cellular responses.

### The PIEZO Receptor and the Carotid Sinus

More specific to the sinus region, the recent evidence produced by Zeng et al. (2018) for the preponderance of PEIZO1 and PEIZO2 receptors in the carotid sinus represents a significant shift in researchers’ understanding the role of mechanotransduction in baroreception. Using a murine model, they injected fluorescent Cholera Toxin B (CTB) underneath the serosa of the sinus region. With the understanding of the location of the baroreceptor cell bodies in the nodose and petrosal ganglia, they quantified the number of CTB-labeled cells that expressed PIEZO1 or PIEZO2 transcripts. Of the total 95 cells that were labeled, six were *PIEZO1* positive, and eight were *PIEZO2* positive. Then they took knock-out mice for both alleles of the PIEZO genes and administered both phenylephrine and sodium nitroprusside. Essentially, the expected baroreceptor reflex was abolished with either drug. Knock-out mice with wild type PIEZO-intact phenotype who were awake and ambulating had a significantly higher average blood pressure and lability with a slightly higher average heart rate than their normal counterparts. This clinical picture is similar to the syndrome of “baroreceptor failure” described in the literature. Given this, the presence of the PIEZO receptor may be fundamental to the function of the carotid sinus.

## Discussion

With the discovery of PIEZO channel aggregation in the sinus region, the carotid sinus may eventually be characterized as a sensory organ in its own right. The uniqueness of the flow in that region combined with the receptor population exquisitely suited to detect it lends itself well to efforts to modulate the action of the carotid sinus to effect cardiovascular changes. The PIEZO receptor family was described by Coste in 2010 and has since been recognized in a remarkable array of cells in which mechanotransduction serves as a nidus for cellular activity. More developments in the PIEZO receptor’s role in baroreceptor mechanism are anticipated.

The variety of organ systems now known to employ the PIEZO channel in converting shear forces into physiologic responses has led to a better understanding of mediators of its activation (Wu et al., 2017a). Wu categorizes the effectors of PIEZO function into those that affect the shear effects on the membrane (i.e., the cell) to which the channel is attached, or the channel itself. Examples of known mediators of PIEZO function that alter the membrane and therefore shear properties include pH, cell hydration, osmotic pressure, and lipid composition. Direct channel mediators include pH, voltage, resting membrane potential, and isolated protein and pharmacologic mediators. For example, the isolation of a molecular agonist named Yoda1 (Syeda et al., 2015), which shortens the inactive state of PIEZO1, raises the possibility that there may be a method by which its inactivation kinetics may be manipulated. These mediators each represent potential methods by which the sinus region can be monitored and altered, and therefore another venue by which carotid sinus effects can be studied.

Furthermore, thanks to advances in researchers’ ability to mine large datasets, biological associations between the PIEZO gene and the carotid sinus can uncover other potential avenues for inquiry. An example of this is Phenoscanner (Staley et al., 2016; Kamat et al., 2019)^1^, with which traits can be cross-referenced with specific genes and gene variants. When examining the PIEZO1 gene, hemodynamically-relevant traits include mean corpuscular volume, hemoglobin concentration, red cell count, whole body water mass, and metabolic rate. It suggests at least a genetic link between blood composition homeostasis and PIEZO-mediated mechanotransduction. As for how and where that transduction of shear force to blood homology takes place, time will tell.

Numerous rheology studies have been conducted to understand the complex properties of blood (Thurston, 1973, 1976, 1979; Chien, 1975; Yeleswarapu, 1996). In Thurston’s work, an oscillating piston cylinder assembly was used to obtain the viscosity curves and showed that blood has both viscoelastic and shear-thinning properties. Blood was centrifuged to disturb any formation between the cells, and then plasma and cells were mixed to ensure certain hematocrit levels. Despite using an oscillatory piston-cylinder system in which fluid cannot experience high residence times–a reflection of the amount of flow recirculation as seen in the sinus–other complex rheological properties were captured in these experiments. In the work by Chien (1975), a Couette viscometer was used to demonstrate the association between hematocrit concentration and viscosity curves. Although more recent studies claimed that non-Newtonian behavior can be neglected in large arteries (Lee and Steinman, 2007; Khan et al., 2017; Arzani, 2018), this assumption for whole blood shall only be valid when the overall shear rate is higher than 100 s^–1^, which is similar to these older rheological experiments (Thurston, 1973, 1976, 1979; Chien, 1975; Yeleswarapu, 1996). Indeed, more recent studies of minimally dissipative CFD schemes have shown the importance of shear-thinning properties in pathological conditions (Bilgi and Atalik, 2019, 2020). An exquisite example of this is the decrease in shear rate with decreased cardiac function in Fontan circulation (Wei H. et al., 2020).

### Understanding the Carotid Sinus to Achieve Homeostatic State and Future Directions

By quantifying the means by which the body “sees” blood *flow*, novel and more precise viscosity biomarkers may one day be available to clinicians. These, in turn, could facilitate care of patients undergoing resuscitation of their blood volume in order to better meet their needs. For example, when reconstituting blood volume lost during surgery, consideration for blood *viscosity* (itself a main component of the shear properties of blood)–in addition to volume and oxygen delivery–may facilitate improved outcomes and more cost-effective administration. Further supporting the sinus region’s possible suitability in this regard, Lee et al. (2020) performed a very elegant study in which they studied changing flow patterns that result from blood *viscosity* after a bolus of crystalloid. To eight healthy subject they administered a one-liter bolus of normal saline. Obtaining viscosity values before and after the bolus, they then simulated the blood flow at the carotid bifurcation and studied the effects on the region of recirculation due to changes in viscosity from the infusion. They observed that the bolus resulted in the accentuation of the shear rate and velocity in the region of recirculation with a measurable decrease in viscosity.

When considering the parameters that affect the activity of the PIEZO receptor and their location, possibly useful endpoints that reflect shear forces caused by blood flow can be studied. One means to approach this is to consider the known mutations in PIEZO genes and the diseases with which they are associated. Any reader with an interest in a more comprehensive review beyond the scope of this piece on physiologic force transduction as mediated by the PIEZO receptor is encouraged to read the review of the current state of knowledge by Beech and Kalli (2019). They include comprehensive descriptions of the PIEZO receptor and its genomic and protein structure and function as it relates to cardiovascular performance. Significantly, they point out that both PIEZO subtypes act as Ca^2+^ ion channels that appear exquisitely sensitive to fluid flows adjacent to cell membranes in which they are incorporated. They go on to suggest that this construct may be used to explain a possible role in the pathophysiology of diseases including lymphatic dysplagia, types of heart failure, hypertension, vascular diseases including aneurysmal ruptures, varicose veins, and anemia.

Looking at the location, morphology, distinctive flow patterns, and PIEZO receptor population of the carotid sinus, we are suggesting that the sinus may be a site of blood viscosity transduction. Given the presence on both the red blood cells and well as the vessel walls, a mutation of the PIEZO receptor may associate comorbid conditions such as anemia with cardiovascular disease that markedly alters blood viscosity–something we see when seeking links in genotype with phenotype. A mutation in the PIEZO receptor that affects the ability to accurately capture the fluid dynamics inside the sinus can alter feedback mechanisms mediated by the autonomic nervous system. This, in turn, can lead to a cardiovascular response that does not appropriately meet the real blood flow and metabolic needs of the patient.

Consider a theoretical mutation in the PIEZO1 receptor where the decrease in blood viscosity with decreased hematocrit is a well-described association (Quemada, 1981). Such a mutation would render a diminished/blunted signal that under normal conditions would correspond with a higher viscosity which would be perceived as a higher hematocrit when, in fact, it is normal or low. In order to increase sinus recirculation and decrease perceived viscosity (Perktold et al., 1991), other organ systems including the renal system, hepatic system, and cardiovascular system would act to retain free water, reduce the viscosity of blood, and increase blood velocity in order to reacquire a “normal” value.

In patients with aberrant blood viscosity of various etiologies, optimization of rheologic parameters, in addition to titrating to pressures, may enable more patient-specific management of blood flow. When trying to understand the physiological machinery the human body uses to detect and maintain hematologic homeostasis, clinicians may better mimic what the autonomic nervous system does to optimize viscosity and, therefore, perfusion. In emulating the apparent physiology utilized at the sinus to characterize blood’s viscometric and shear stress properties, we can design devices that better aid management of patients with compromised blood delivery, or mitigate the effects pathophysiologic shear patterns have on the carotid vessel walls. Certainly, the fact that the location of the carotid vessels in the neck lends itself to easy visualization by non-invasive methods, including ultrasound, facilitates this. By using parameters like sinus geometry and blood velocity that can be easily obtained by a Doppler scan, recent machine learning algorithms based on deep learning can be used to guide physicians. Administration of blood products, blood expanders, and other agents to either increase or decrease blood viscosity can be titrated to specific WSS parameters and, ultimately, affect viscosity homeostasis.

## Author Contributions

AI and CB contributed to the conceptualization, writing (original draft), manuscript review, and editing. RN, TB, DN, and NP contributed to conceptualization, manuscript review, and editing. All authors contributed to the article and approved the submitted version.

## Conflict of Interest

The authors declare that the research was conducted in the absence of any commercial or financial relationships that could be construed as a potential conflict of interest.

## Footnotes

1 http://www.phenoscanner.medschl.cam.ac.uk/
